# Supplementary material for: Optogenetic modulation of TDP-43 oligomerization accelerates ALS-related pathologies in the spinal motor neurons
Source: Nat Commun. 2020 Feb 21;11:1004. doi: 10.1038/s41467-020-14815-x (PMC7035286; doi:10.1038/s41467-020-14815-x)
Supplement: Supplementary file 1 — Supplementary Information [file 41467_2020_14815_MOESM1_ESM.pdf]

Supplementary Information

Optogenetic modulation of TDP-43 oligomerization accelerates ALS-related pathologies in the spinal motor neurons

(Asakawa et al.)

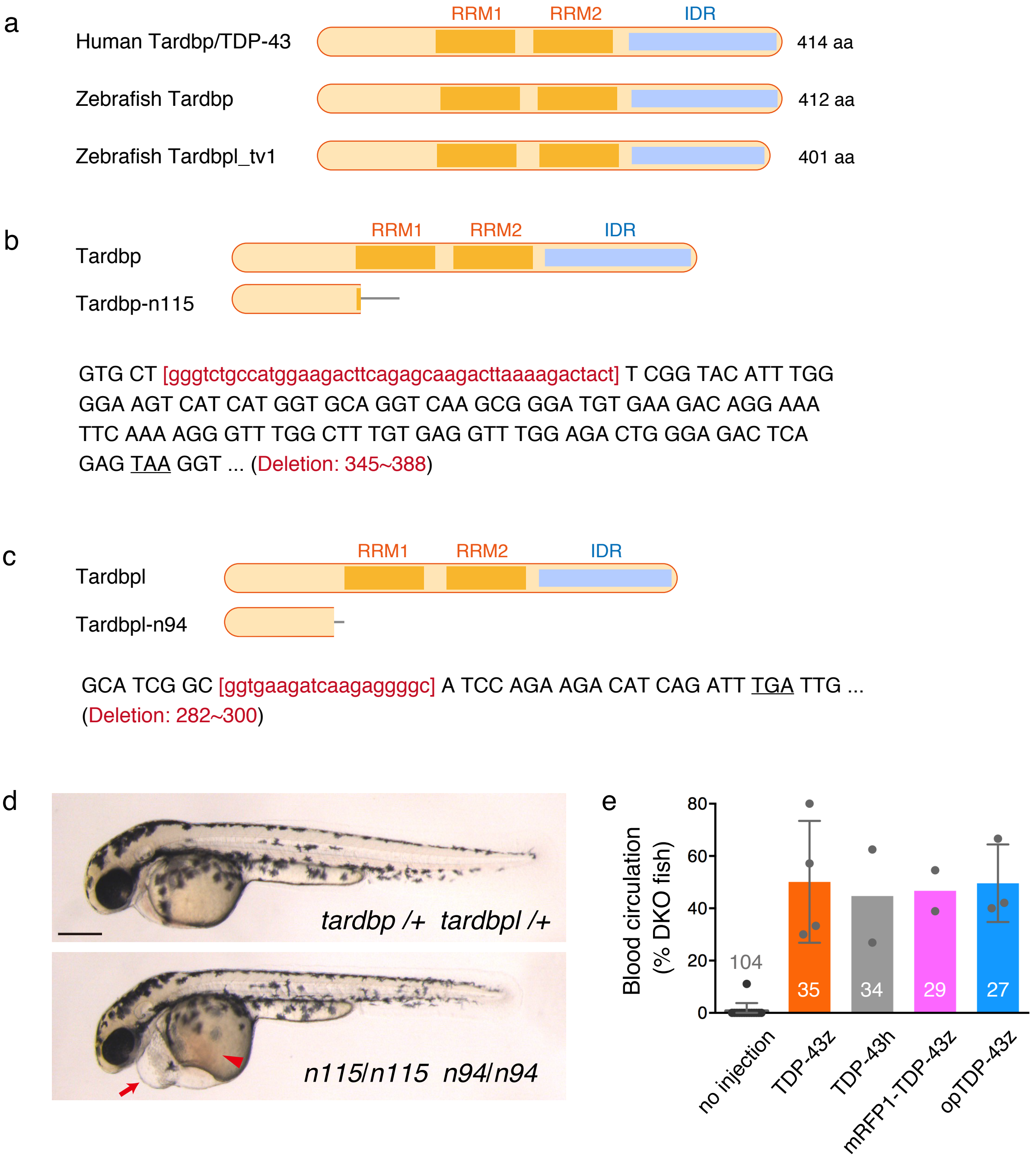

Supplementary Figure 1

Functional validation of opTDP-43z using TDP-43 knockout fish.

(a) The structure of human and zebrafish Tardbp/TDP-43 proteins. RRM, RNA-recognition motif. IDR, intrinsically disordered region. (b, c) The tardbp-n115 and tardbpl-n94 mutations are frame-shift deletions that cause protein truncation. The deleted nucleotides were indicated by red lower cases. The grey bars indicate ectopically added peptide due to the frame shift. (d) The lateral views of the wild type (top) and double homozygous (bottom) larvae at 48 hpf. The arrow and arrowhead indicate the swollen heart and the stacked red blood cells on the far side of the yolk, respectively. The bar indicates 250 μm. (e) Rescue rate of the blood circulation defect of the tardbp-n115 tardbpl-n94 homozygotes (DKOs). The numbers on the histograms show the total numbers of DKOs investigated (Source data are provided as a Source Data file). Error bars show SD.

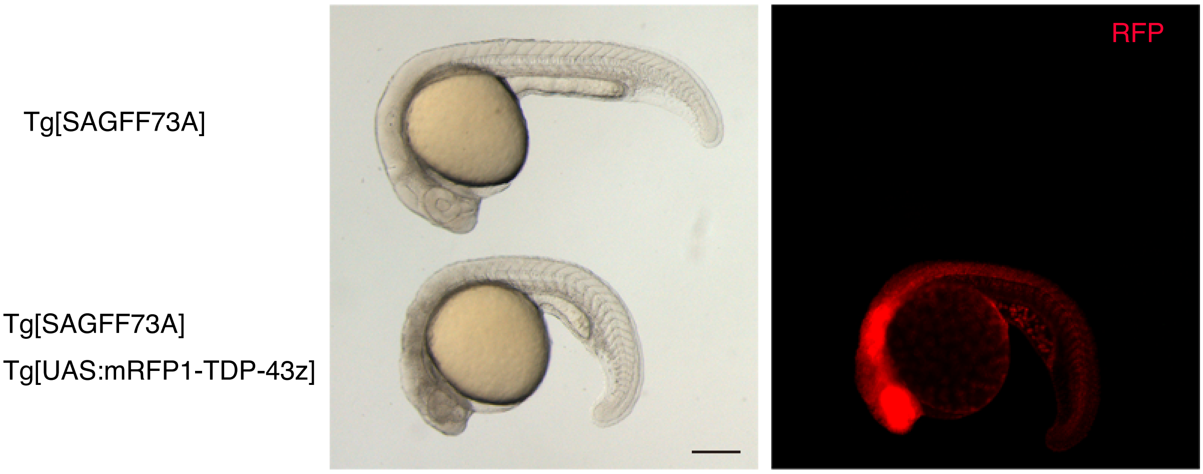

Supplementary Figure 2

Whole-body overexpression of mRFP1-TDP-43z is toxic to zebrafish.

The lateral view of Tg[SAGFF73] (top) and Tg[SAGFF73] Tg [UAS:mRFP1-TDP-43z] (bottom) embryos at 24 hpf. The RFP signal is shown in the right panel. The bar indicates 250 μm.

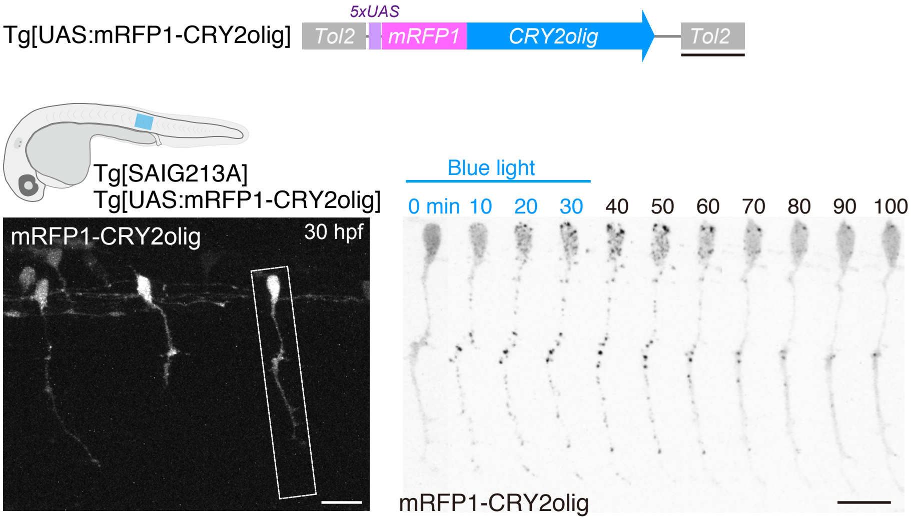

Supplementary Figure 3

Rapid and reversible clustering of mRFP1-CRY2olig in the spinal motor neurons.

The lateral view of CaPs of Tg[SAIG213A] Tg[UAS:mRFP1-CRY2olig] double transgenic embryo. The blue light was illuminated from 0 to 30 min. The bar indicates 20 μm.

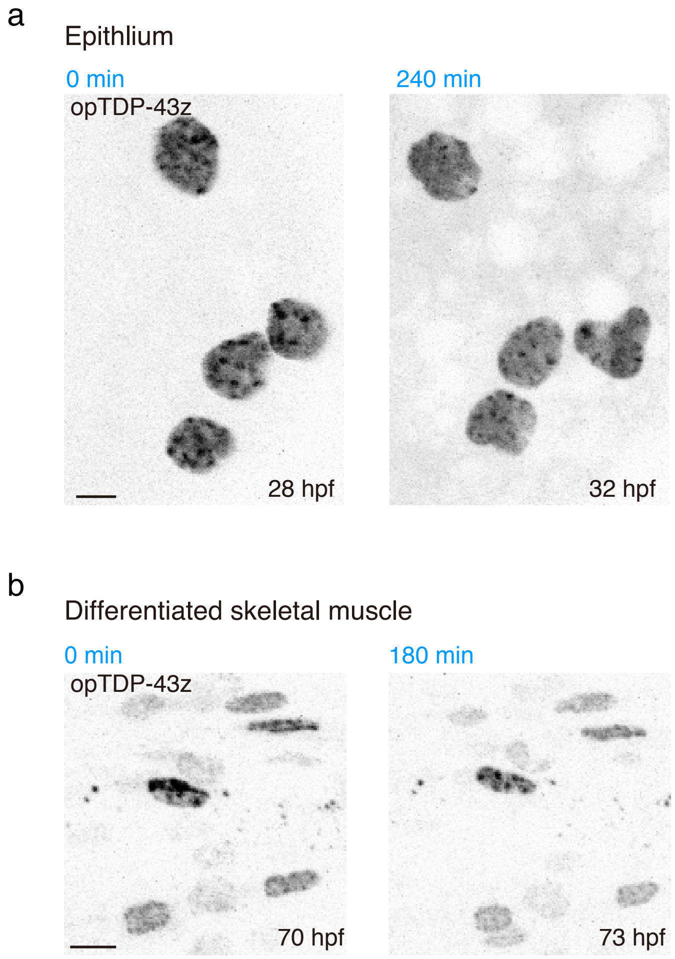

Supplementary Figure 4

Cell-type specificity of light-dependent cytoplasmic opTDP-43z mislocalization.

(a) The nuclear-enriched opTDP-43z localization was not changed by the 3-hour blue light illumination in the embryonic epithelial cells. (b) opTDP-43z localization was not changed by the 4-hour blue light illumination in the differentiated skeletal muscle cells. Cytoplasmic opTDP-43 foci were occasionally observed independently of light illumination. These light-insensitive opTDP-43 foci were static throughout the experiment. The bar indicates 10 μm.

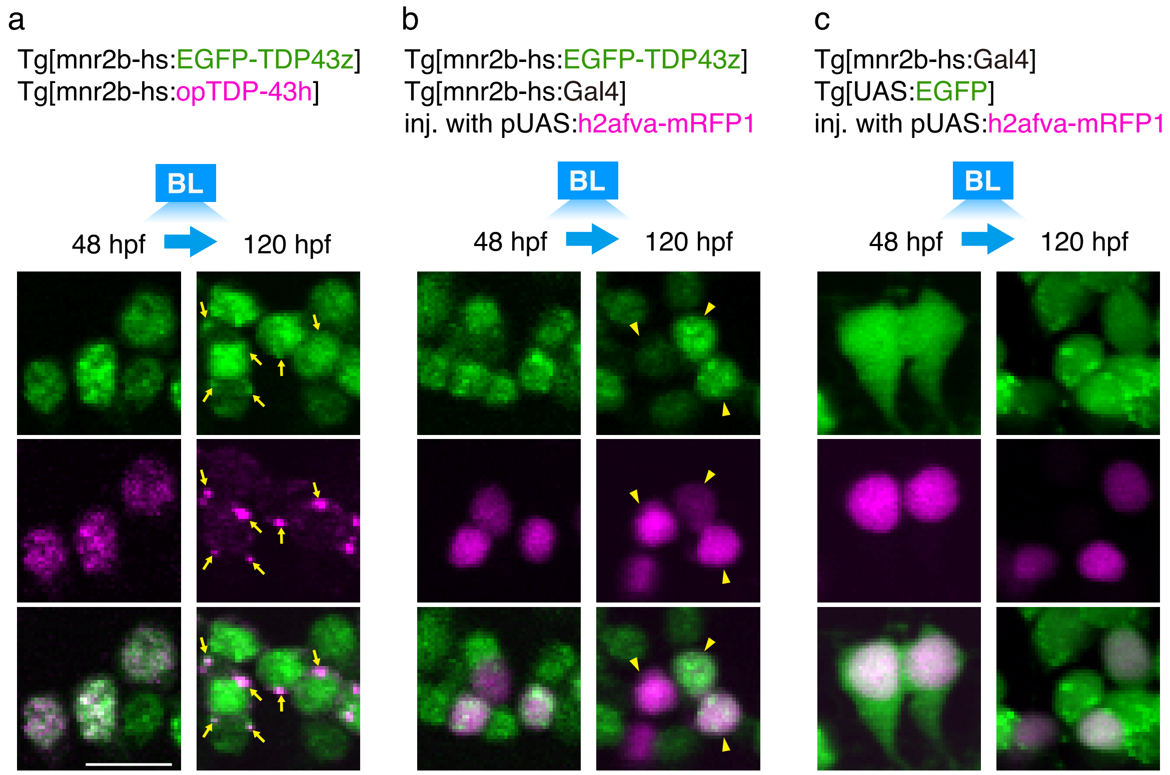

Supplementary Figure 5

Blue light illumination alone does not cause cytoplasmic mislocalization of EGFP-TDP-43z.

(a) opTDP-43h forms cytoplasmic aggregates containing EGFP-TDP-43z by blue light illumination (BL) during 48 - 120 hpf (arrows). (b) EGFP-TDP-43z displays nuclear localization after the illumination, as judged by the nuclear marker H2afv2-mRFP1 expressed from plasmid pUAS:h2afva-mRFP1 injected at the one-cell stage. (c) Visualization of the nucleus of spinal motor neuron by the fluorescent histone H2A vaiant, H2afv2-mRFP1. In all panels, the cells at 48 hpf and 120 hpf are independent. The bar indicates 10 μm.

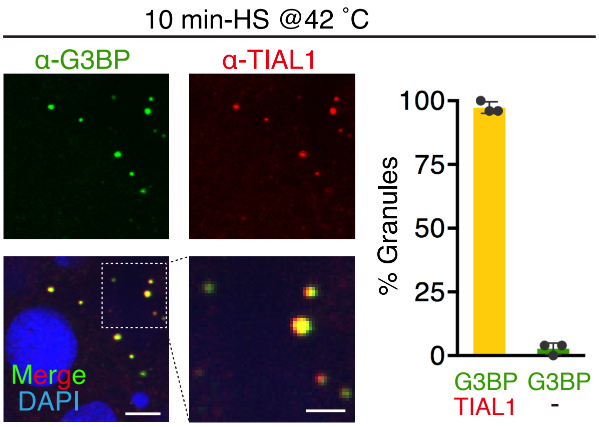

Supplementary Figure 6

Human antibodies against G3BP and TIAL1 recognize heat-shock induced SGs in zebrafish

Anti-G3BP and anti-TIAL1 antibodies nearly completely colocalize with each other in the cytoplasmic granules induced by heat shock (42 ˚C for 10 minutes) in the epithelial cells of zebrafish embryos at 24 hpf. At least, twenty distinct cytoplasmic granules labeled by anti-G3BP were examined for anti-TIAL1 immunoreactivity in each of 3 independent fish. The bars indicate 5 μm (left) and 2 μm (right).
