# Supplementary material for: Optogenetic modulation of TDP-43 oligomerization accelerates ALS-related pathologies in the spinal motor neurons
Source: Nat Commun. 2020 Feb 21;11:1004. doi: 10.1038/s41467-020-14815-x (PMC7035286; doi:10.1038/s41467-020-14815-x)
Supplement: Supplementary file 3 — Description of Additional Supplementary Files [file 41467_2020_14815_MOESM3_ESM.pdf]

Description of Additional Supplementary Files

File Name: Supplementary Movie 1
Description: 1 Rescue experiments of blood circulation defect of TDP-43 DKO. Arrows indicate the stream of blood cells on the yolk surface. Note that TDP-43 DKOs (n115/n115, n94/n94) do not exhibit flow of blood cells, and the red blood cells are stacked (arrowhead).

File Name: Supplementary Movie 2
Description: The skeletonized CaP axons of Tg[SAIG213A] Tg[UAS:EGFP] (left) and Tg[SAIG213A] Tg[UAS:EGFP] Tg[UAS:mRFP1-TDP-43z] (right). The axon branch points and terminals are indicated by red and green, respectively.

File Name: Supplementary Movie 3
Description: Skeletal muscle cells expressing opTDP-43z opTDP-43z illuminated with blue light in in Tg[SAGFF73A] Tg[UAS:opTDP-43z] fish at 28 hpf.

File Name: Supplementary Movie 4
Description: The lateral view of CaPs of Tg[SAIG213A] Tg[UAS:mRFP1-CRY2olig] double transgenic embryo. The blue light was illuminated from 0 to 30 min (indicated by a blue dot).

File Name: Supplementary Movie 5
Description: The dorsal view of the spinal cord at the segment 14 -17 levels of a Tg[SAIG213A] Tg[mnr2b-hs:Gal4] Tg[UAS:opTDP-43z] Tg[UAS:EGFP] quadruple transgenic fish. EGFP (left) and opTDP-43z (right) were presented side by side. The transverse planes for EGFP and opTDP-43z signals are not completely matched because they are independently registered using Image J software.

File Name: Supplementary Movie 6
Description: A Tg[mnr2b-hs:EGFP-TDP43z] Tg[mnr2b-hs:opTDP-43hA315T] (A315T) larva (focused) that had been illuminated with blue light during 48 - 120 hpf is lying at the bottom of the dish with normal heart beat but without the swim bladder inflation. Its sibling (Tg[mnr2b-hs:EGFP-TDP43z] larva) that experienced the same illumination procedure is capable of swimming freely with an inflated swim bladder. The movie is played in actual speed.
