# Supplementary material for: Optogenetic modulation of TDP-43 oligomerization accelerates ALS-related pathologies in the spinal motor neurons
Source: Nat Commun. 2020 Feb 21;11:1004. doi: 10.1038/s41467-020-14815-x (PMC7035286; doi:10.1038/s41467-020-14815-x)
Supplement: Supplementary file 10 — Reporting summary [file 41467_2020_14815_MOESM10_ESM.pdf]

nature research

Corresponding author(s): Kazuhide Asakawa
Koichi Kawakami

Last updated by author(s): 30th, Jan, 2020

Reporting Summary

Nature Research wishes to improve the reproducibility of the work that we publish. This form provides structure for consistency and transparency in reporting. For further information on Nature Research policies, see Authors & Referees and the Editorial Policy Checklist.

Statistics

For all statistical analyses, confirm that the following items are present in the figure legend, table legend, main text, or Methods section.

n/a	Confirmed	
☐	☒	The exact sample size (*n*) for each experimental group/condition, given as a discrete number and unit of measurement
☐	☒	A statement on whether measurements were taken from distinct samples or whether the same sample was measured repeatedly
☐	☒	The statistical test(s) used AND whether they are one- or two-sided<br>*Only common tests should be described solely by name; describe more complex techniques in the Methods section.*
☒	☐	A description of all covariates tested
☒	☐	A description of any assumptions or corrections, such as tests of normality and adjustment for multiple comparisons
☐	☒	A full description of the statistical parameters including central tendency (e.g. means) or other basic estimates (e.g. regression coefficient) AND variation (e.g. standard deviation) or associated estimates of uncertainty (e.g. confidence intervals)
☐	☒	For null hypothesis testing, the test statistic (e.g. *F*, *t*, *r*) with confidence intervals, effect sizes, degrees of freedom and *P* value noted<br>*Give P values as exact values whenever suitable.*
☒	☐	For Bayesian analysis, information on the choice of priors and Markov chain Monte Carlo settings
☒	☐	For hierarchical and complex designs, identification of the appropriate level for tests and full reporting of outcomes
☒	☐	Estimates of effect sizes (e.g. Cohen's *d*, Pearson's *r*), indicating how they were calculated

Our web collection on statistics for biologists contains articles on many of the points above.

Software and code

Policy information about availability of computer code

Data collection	Image data were collected with Olympus FV1200 microscope operated by the software supplied by Olympus.
Data analysis	Image Data were analyzed with Olympus Fluoview Ver2.1b Viewer, Image J (2.0.0-rc-43/1.52n), GraphPad Prism 6. Adobe photoshop CS6 was used for figure presentation.

For manuscripts utilizing custom algorithms or software that are central to the research but not yet described in published literature, software must be made available to editors/reviewers. We strongly encourage code deposition in a community repository (e.g. GitHub). See the Nature Research guidelines for submitting code & software for further information.

Data

Policy information about availability of data

All manuscripts must include a data availability statement. This statement should provide the following information, where applicable:
- Accession codes, unique identifiers, or web links for publicly available datasets
- A list of figures that have associated raw data
- A description of any restrictions on data availability

All data generated or analyzed during this study are included in this published article (and its supplementary information files) or available from Kazuhide Asakawa on reasonable request.

Field-specific reporting

Please select the one below that is the best fit for your research. If you are not sure, read the appropriate sections before making your selection.

☒ Life sciences ☐ Behavioural & social sciences ☐ Ecological, evolutionary & environmental sciences

Life sciences study design

All studies must disclose on these points even when the disclosure is negative.

Sample size	The sample size for CaP morphological analyses was determined on the observation that the axon lengths of CaPs in the wild type were judged as normal by Shapiro-Wilk Normality Test. The sample size was set in a similar range in other experiments.
Data exclusions	No data was excluded.
Replication	Data were obtained at least from three independent animals for each condition, except that data was obtained from 2 independent animal in Figure 5e (mRFP1-CRY2olig). All attempts at replication were successful. Experimental genetic materials used in this study have been kept in the lab or can be reproduced if necessary.
Randomization	Randomization was not relevant to this study because we have treated the experimental samples equally (e.g. raising fish in the same well of plastic dishes), except during individual imaging under the microscope. The imaging order did not affect the results.
Blinding	For the light illumination experiments against individual fish, we needed to select fish carrying appropriate fluorescent proteins from multiple transgenes. Therefore, complete blinding in data collection was not possible. Our key findings were associated with experimental manipulations such as specific genetic manipulation and/or light illumination, but not with the order of data collection or analysis.

Reporting for specific materials, systems and methods

We require information from authors about some types of materials, experimental systems and methods used in many studies. Here, indicate whether each material, system or method listed is relevant to your study. If you are not sure if a list item applies to your research, read the appropriate section before selecting a response.

Materials & experimental systems

n/a	Involved in the study	
☐	☒	Antibodies
☒	☐	Eukaryotic cell lines
☒	☐	Palaeontology
☐	☒	Animals and other organisms
☒	☐	Human research participants
☒	☐	Clinical data

Methods

n/a	Involved in the study	
☒	☐	ChIP-seq
☒	☐	Flow cytometry
☒	☐	MRI-based neuroimaging

Antibodies

Antibodies used

1, Mouse monoclonal antibody for mono- and polyubiquitinate conjugates (Clone:FK2, Enzo)
2, Goat anti-mouse IgG Alexa Fluor 488 (A11001, Molecular Probes)
3, Rabbit anti-RFP polyclonal antibody (pAb, MBL)
4, Goat anti-rabbit IgG Alexa Fluor 633 (A21070, Molecular Probes)
5, Mouse anti-human G3BP (Clone:23/G3BP, BD Transduction Laboratories)
6, Rabbit anti-TIAL1 antibody (NBP1-79932, Novus Biologicals)
7, Mouse anti-phospho TDP-43 (pS409/410) (Clone:11-9, TIP-PTD-MO1, Cosmo Bio)
8, Goat anti-rabbit IgG Alexa Fluor 488 (A11008, Molecular Probes)

Validation

1, http://www.enzolifesciences.com/BML-PW8810/mono-and-polyubiquitinylated-conjugates-monoclonal-antibody-fk2/
2, https://www.thermofisher.com/antibody/product/Goat-anti-Mouse-IgG-H-L-Secondary-Antibody-Oligoclonal/A28175
3, http://ruo.mbl.co.jp/bio/dtl/A/?pcd=PM005
4, https://www.thermofisher.com/antibody/product/Goat-anti-Rabbit-IgG-H-L-Cross-Adsorbed-Secondary-Antibody-Polyclonal/A-21070
5, https://www.bdbiosciences.com/eu/reagents/research/antibodies-buffers/cell-biology-reagents/cell-biology-antibodies/purified-mouse-anti-human-g3bp-23g3bp/p/611127
6, https://www.novusbio.com/products/tial1-antibody_nbp1-79932
7, https://search.cosmobio.co.jp/cosmo_search_p/search_gate2/docs/CAC_/TIPPTDM01.20130226.pdf
8, https://www.thermofisher.com/antibody/product/Goat-anti-Rabbit-IgG-H-L-Cross-Adsorbed-Secondary-Antibody-Polyclonal/A-11008

Animals and other organisms

Policy information about studies involving animals; ARRIVE guidelines recommended for reporting animal research

Laboratory animals	This study used the zebrafish (Danio rerio) , which is a hybrid of AB and TL, with both sexes, and embryos and larvae obtained from them.
Wild animals	This study did not involve wild animals.
Field-collected samples	This study did not involve samples collected from the field.

Ethics oversight	This study was carried out in accordance with the Guide for the Care and Use of Laboratory Animals of the Institutional Animal Care and Use Committee (IACUC, approval identification number 24-2) of the National Institute of Genetics (NIG, Japan), which has an Animal Welfare Assurance on file (assurance number A5561-01) at the Office of Laboratory Animal Welfare of the National Institutes of Health (NIH, USA).

Note that full information on the approval of the study protocol must also be provided in the manuscript.
